# Comparative Shotgun Metagenomics of Microbial Communities Associated with Two Geothermal Systems in Kazakhstan

**DOI:** 10.3390/biology15191704

**Published:** 2026-09-24

**Authors:** Ardak B. Kakimova, Yelena G. Zaparina, Bolatkhan K. Zayadan, Sandugash K. Sandybayeva, Dinara Kelden, Aliyam A. Dauletova, Silvano Piazza

**Affiliations:** 1 Department of Chemical and Biological Technologies, K. Turysov Institute of Geology and Oil and Gas Engineering, Satbayev University, 22 Satpaev Street, Almaty 050013, Kazakhstan; sandybaevasandugash@gmail.com (S.K.S.); dinarkeldenkyzy@gmail.com (D.K.); 2 Department of Biotechnology, Faculty of Biology and Biotechnology, Al-Farabi Kazakh National University, Al-Farabi 71, Almaty 050038, Kazakhstan; zbolatkhan@gmail.com (B.K.Z.); dauletova.aliyam13@gmail.com (A.A.D.); 3 Department of Botany and Agroecology, Faculty of Biology and Biotechnology, Al-Farabi Kazakh National University, Al-Farabi 71, Almaty 050038, Kazakhstan; 4 Computational Biology, International Centre for Genetic Engineering and Biotechnology, ICGEB, 34149 Trieste, Italy; piazza@icgeb.org

**Keywords:** shotgun metagenomics, geothermal systems, microbial communities, taxonomic diversity, functional potential, hydrogen metabolism, Kazakhstan

## Abstract

Geothermal-associated microbial communities remain poorly characterized in Central Asia. Here, shotgun metagenomics was used to compare microbial communities associated with thermally influenced substrates from the Chundzha and Rakhman geothermal systems in Kazakhstan. Six metagenomes representing three spatial replicates per site yielded approximately 6.5–7.3 Gb of clean sequence data per sample. Species-level richness showed a directional trend toward higher values in Chundzha, whereas Shannon diversity was similar between sites. Genus-level Bray–Curtis ordination showed apparent site-associated separation, with sampling site explaining 87.3% of the variation, although the effect was not statistically significant (PERMANOVA, R^2^ = 0.873, *p* = 0.10). Chundzha showed greater relative representation of several halophilic and halotolerant taxa, whereas Rakhman showed greater representation of soil-associated lineages. Broad COG and KEGG functional profiles were largely shared between sites. Several hydrogenase-, nitrogenase-, and RuBisCO-associated genes showed higher relative representation in Rakhman, whereas selected fermentation- and photosynthesis-associated functions were relatively higher in Chundzha; however, none of the targeted KO comparisons remained significant after FDR correction. These results indicate candidate taxonomic and functional patterns that should be regarded as exploratory because of the limited replication. The dataset provides a baseline for future genome-resolved and metatranscriptomic studies of geothermal-associated microbial communities in Kazakhstan.

## Simple Summary

Geothermal systems can support diverse microbial communities with potentially useful metabolic capabilities. However, such environments remain poorly characterized in Kazakhstan. In this study, we compared microbial communities associated with the Chundzha and Rakhman geothermal systems using shotgun metagenomics. Chundzha showed a directional trend toward higher species richness, whereas Shannon diversity was similar between the two sites. Genus-level community profiles showed apparent separation, with sampling site explaining 87.3% of the variation in Bray–Curtis space, although this effect was not statistically significant (PERMANOVA, *p* = 0.10). Broad functional profiles were largely shared between sites. Rakhman showed higher relative representation of several genes associated with hydrogen metabolism, nitrogen fixation, and RuBisCO-related carbon fixation, whereas Chundzha showed higher representation of selected fermentation- and photosynthesis-associated functions. None of the targeted KO comparisons remained significant after false discovery rate correction. These findings identify candidate taxonomic and metabolic patterns for further investigation in geothermal-associated microbial communities of Kazakhstan.

## 1. Introduction

Geothermal springs are naturally heterogeneous ecosystems in which temperature, pH, mineral composition, redox conditions, and the availability of electron donors and acceptors act as environmental filters on microbial communities [1,2]. These gradients can generate pronounced differences in community composition even among geographically related thermal systems, making geothermal habitats useful models for examining links between environmental conditions, microbial diversity, and metabolic potential [3].

Large-scale and cross-system surveys indicate, however, that the relative importance of individual environmental drivers is context-dependent. Across 925 geothermal springs in New Zealand, pH was the strongest correlate of microbial diversity below approximately 70 °C, whereas temperature became more influential at higher temperatures [4]. By contrast, a survey spanning 36 geothermal areas in Canada and New Zealand reported a strong temperature–diversity relationship across a broad thermal range [5]. Studies of Tibetan Plateau hot springs likewise showed that temperature strongly structured community composition even when richness and Shannon diversity were not significantly correlated with temperature [6], while Great Boiling Spring demonstrated strong temperature effects in sediments and clear differentiation between sediment- and water-associated communities [7]. Additional work in alkaline Yellowstone springs showed temperature-associated shifts in bacterial and archaeal assemblages [8], and metagenomic studies of Trans-Himalayan and Spanish thermal systems linked taxonomic and functional patterns to local geochemical conditions [9,10]. Together, these findings argue against a single universal environmental driver and support comparative designs that evaluate both community composition and functional potential.

Culture-independent sequencing has greatly expanded knowledge of geothermal microbiomes. Shotgun metagenomics is particularly informative because taxonomic composition and functional gene repertoires can be characterized from the same dataset without prior cultivation. Studies of terrestrial hot springs have revealed diverse bacterial and archaeal assemblages together with genes involved in energy metabolism, carbon fixation, nitrogen cycling, stress adaptation, and other processes relevant to survival under extreme conditions [11,12]. Recent genome-resolved surveys have further shown that geothermal ecosystems contain extensive uncultured taxonomic and metabolic diversity that is poorly represented in isolate collections [12]. Recent large-scale and regional sequencing surveys further illustrate substantial spatial heterogeneity in hot-spring microbial communities and functional repertoires across contrasting physicochemical settings [13,14].

Among the functions of particular ecological and biotechnological interest are hydrogen metabolism, nitrogen fixation, and autotrophic carbon assimilation. Hydrogenases are widespread in geothermal microorganisms and can support hydrogen oxidation, hydrogen evolution, or other redox processes depending on enzyme class and physiological context [2,15]. Biological nitrogen fixation has been demonstrated in high-temperature geothermal communities, and nif-associated genes occur across diverse thermophilic lineages [16,17]. Carbon fixation is likewise distributed among phylogenetically distinct geothermal microorganisms and may proceed through several autotrophic pathways [18,19]. These functions are also relevant to bioenergy and carbon-utilization research, although metagenomic detection indicates genomic potential rather than pathway activity [20].

Compared with intensively investigated geothermal regions in North America and Asia, Central Asian thermal ecosystems remain comparatively underrepresented in shotgun metagenomic studies. A recent metagenomic analysis of a high-altitude geothermal spring in Tajikistan recovered previously unresolved thermophilic archaeal diversity and multiple hydrogenase systems [15]. In Kazakhstan, metagenomic and culture-based analysis of the Zharkent geothermal spring identified diverse thermophilic bacterial populations [21], while cyanobacterial communities have been reported from hot springs in the Almaty region, including Chundzha-associated environments [22]. However, comparative shotgun metagenomic studies of geographically separated geothermal systems within Kazakhstan remain scarce.

The Chundzha and Rakhman geothermal systems provide a useful comparison because they occur in contrasting geographic and hydrogeological settings. Chundzha is associated with low-salinity geothermal groundwater of the southeastern Ili Basin [23], whereas the Rakhman springs occur in the mountainous Southern Altai and have been described as siliceous, radon-bearing thermal waters [24]. Such contrasting settings provide an ecological context for examining whether thermally influenced substrate communities differ in taxonomic composition while retaining overlapping genomic functional potential. Because complete physicochemical measurements were not available for each metagenomic replicate, these environmental contrasts are treated as contextual hypotheses rather than sample-level explanatory variables.

The study aimed to compare the taxonomic composition and genomic functional potential of microbial communities associated with thermally influenced substrates from the Chundzha and Rakhman geothermal systems in Kazakhstan. Accordingly, we further evaluated candidate functions related to hydrogen metabolism, nitrogen fixation, RuBisCO-associated carbon fixation, lipid metabolism, fermentation, and photosynthesis as targets for future genome-resolved and expression-based investigation.

## 2. Materials and Methods

### 2.1. Study Sites and Sample Collection

Environmental samples were collected in July 2025 from two geographically separated geothermal systems in Kazakhstan: the Chundzha thermal spring area in southeastern Kazakhstan and the Rakhman geothermal springs in eastern Kazakhstan. The analyzed material consisted of moist soil/substrate directly exposed to thermal water rather than ambient terrestrial soil collected away from the springs. Sampling locations, coordinates, elevations, and sample codes are summarized in Table 1 and Figure 1.

At each site, moist substrate in direct contact with thermal water was collected independently from three spatially separated sampling points using sterile tools, providing three spatial replicates per site. Replicate points were located approximately 5 m apart. The Chundzha samples were designated C1_Met_1, C1_Met_2, and C1_Met_3, and the Rakhman samples U1_Met_1, U1_Met_2, and U1_Met_3. During sampling, thermal-water temperature at Chundzha ranged from approximately 38 to 50 °C with pH 7–8, whereas Rakhman thermal waters were approximately 30–40 °C with a pH of about 8. Samples were transferred to sterile containers, transported to the laboratory at approximately 4 °C, and stored at −20 °C until DNA extraction. Additional details on the field sampling framework, sampled matrix, spatial replicate design, aseptic sample handling, and transport and storage conditions are provided in Supplementary Method S1.

Chundzha is associated with weakly mineralized thermal groundwater of the southeastern Ili Basin [23], whereas the Rakhman springs occur in the mountainous Southern Altai and are associated with radon-bearing thermal waters [24]. These literature-derived hydrochemical characteristics are provided as environmental background only. Because matched measurements of salinity, conductivity, major ions, and other geochemical variables were not available for all six metagenomic replicates, such variables were not included as explanatory covariates in the statistical analyses.

### 2.2. DNA Extraction, Shotgun Sequencing, and Metagenomic Processing

Total community DNA was extracted from the moist soil/substrate samples using the E.Z.N.A.^®^ Soil DNA Kit (Omega Bio-tek, Norcross, GA, USA) according to the manufacturer’s instructions and assessed by 1% agarose gel electrophoresis. Genomic DNA was fragmented using a Covaris M220 instrument (Covaris, LLC, Woburn, MA, USA), followed by paired-end library preparation including adapter ligation, magnetic-bead size selection, PCR enrichment, and library denaturation. Shotgun metagenomic sequencing was performed by Majorbio Bio-Pharm Technology Co., Ltd. (Shanghai, China) on an Illumina NovaSeq platform (Illumina, Inc., San Diego, CA, USA) using paired-end 150 bp sequencing. Downstream bioinformatic processing was conducted using the Majorbio Cloud Platform (Majorbio Bio-Pharm Technology Co., Ltd., Shanghai, China; https://www.majorbio.com/tools, accessed on 22 August 2026) following the provider’s metagenomic workflow.

Raw reads were quality-filtered using fastp v0.23.0 [25] and assembled independently with MEGAHIT v1.2.9 [26]. Contigs shorter than 500 bp were excluded. Open reading frames were predicted using Prodigal v2.6.3 [27], and predicted sequences shorter than 100 bp were removed. Genes were clustered with CD-HIT v4.6.1 [28] at ≥90% sequence identity and ≥90% coverage to generate a non-redundant gene catalogue.

Clean reads were mapped back to the non-redundant gene catalogue using SOAPaligner v2.21 at ≥95% identity for gene-abundance estimation. This threshold corresponds to the Majorbio read-to-catalogue abundance-mapping workflow and was not used for species-level taxonomic classification. Because a 95% identity cutoff may permit mapping among closely related homologous catalogue genes, the resulting abundance estimates may be somewhat broader than those obtained with a more stringent cutoff. A separate sensitivity remapping at 97% identity was not performed; this potential source of abundance uncertainty was therefore considered when interpreting gene-level differences.

### 2.3. Taxonomic and Functional Annotation

Predicted proteins were aligned against the NCBI non-redundant protein database (NR; release 20251204_v3) using DIAMOND v2.0.13 in BLASTP mode [29] with an E-value threshold of ≤1 × 10^−5^. Taxonomic assignments were obtained using the Majorbio Best-hit method based on the DIAMOND-NR results and summarized from domain to species level. Genus-level RPKM-normalized taxonomic abundances were used for community-composition and beta-diversity analyses. Differential taxonomic signatures were evaluated using linear discriminant analysis effect size (LEfSe) [30]. Because only three spatial replicates were available per site and no FDR correction was applied within the LEfSe feature set, the LEfSe analysis was treated as exploratory. Features with nominal *p* < 0.05 and LDA > 2.0 were retained in the complete output, while genus-level features with LDA > 4.0 were emphasized in the main-text figure.

Functional annotation was performed against KEGG (release 20241007) [31], COG (2020 release) [32], and MetaCyc (v201901). KEGG assignments were summarized at Pathway Levels 2 and 3 and at the KEGG Orthology (KO) level. Broad taxonomic community analyses used genus-level RPKM abundance profiles, whereas targeted KO comparisons used TPM-normalized KO abundances. COG category comparisons were based on mapped gene-abundance counts generated by the Majorbio workflow. Thus, a single specified normalization method was used within each downstream comparison rather than an interchangeable “RPKM or TPM” procedure.

Targeted KO analysis focused on genes associated with hydrogen metabolism and hydrogenase maturation (hup/hyp), nitrogen fixation (nif), RuBisCO-associated carbon fixation (rbc), lipid metabolism (acc, fab, and pls), fermentation, and selected photosynthesis-associated functions. *rbcL* and *rbcS* were included as representative markers of the Calvin–Benson–Bassham cycle and were not intended to provide comprehensive coverage of all autotrophic carbon-fixation pathways. TPM-normalized KO abundances were compared between sites using two-tailed Wilcoxon rank-sum tests. FDR adjustment was applied across the complete targeted KO set included in the comparison. Detected genes were interpreted as genomic functional potential rather than evidence of transcriptional activity, pathway completeness, or metabolic flux.

### 2.4. Diversity and Statistical Analysis

Alpha diversity was calculated at the species level from NR-based taxonomic profiles using read-number abundance data in the Majorbio Cloud Platform. Observed richness (Sobs), abundance-based coverage estimator (ACE), Chao1 richness, and Shannon diversity were calculated. No user-defined abundance filtering threshold was applied in the alpha-diversity module. For the present samples, the species-level profiles contained no singleton species (n1 = 0); under the platform’s Chao1 and ACE implementations this yields no unseen-richness correction, resulting in Sobs = Chao1 = ACE for each sample. Differences between Chundzha and Rakhman were evaluated using two-tailed Wilcoxon rank-sum tests, with FDR correction applied across the tested alpha-diversity metrics.

For beta-diversity analysis, Bray–Curtis dissimilarities were calculated at the genus level from RPKM-normalized taxonomic abundance profiles. Principal coordinates analysis (PCoA) and non-metric multidimensional scaling (NMDS) were used for visualization. Between-site differences were evaluated using analysis of similarities (ANOSIM) [33] and PERMANOVA (Adonis) [34] with 999 permutations. Homogeneity of multivariate dispersion was assessed using PERMDISP [35] on the same Bray–Curtis dissimilarity matrix, based on distances to group centroids.

Accordingly, statistical analyses and visualization were performed using the Majorbio Cloud Platform and associated R-based workflows, with the PERMDISP check calculated from the exported genus-level RPKM abundance matrix. The comparison included three spatial replicates per site (*n* = 3). A two-sided *p* < 0.05 was considered statistically significant, and FDR-adjusted *q*-values were used where applicable following the Benjamini–Hochberg procedure [36]. The NMDS platform output reported stress = 0.000; because the interface does not expose the number of random starts or other optimization settings, this fit metric was interpreted cautiously and was not treated as independent evidence of significant community differentiation.

No custom computer code or software was developed for this study. Bioinformatic processing and statistical analyses were performed using the software packages and Majorbio Cloud Platform workflows described above.

## 3. Results

### 3.1. Metagenomic Dataset Characteristics

Shotgun metagenomic sequencing was performed for three spatial replicates from each geothermal site, comprising six samples in the Chundzha–Rakhman comparison. The Chundzha samples were designated C1_Met_1, C1_Met_2, and C1_Met_3, and the Rakhman samples U1_Met_1, U1_Met_2, and U1_Met_3.

The six libraries generated approximately 43.49–48.70 million raw reads per sample, corresponding to 6.57–7.35 Gb of raw sequence data. Following quality filtering, 43.25–48.41 million reads per sample were retained, representing 99.40–99.49% of the original reads. Clean sequence yield ranged from approximately 6.52 to 7.29 Gb per sample, indicating limited read loss during preprocessing (Table 2).

Assembly statistics varied across the six metagenomes, with 91,171–213,037 contigs per sample and total assembly lengths ranging from 65.55 to 191.94 Mb. N50 values ranged from 680 to 855 bp, N90 values from 527 to 547 bp, and maximum contig lengths from 9390 to 324,201 bp. Gene prediction identified 120,516–322,892 ORFs per sample, with mean predicted ORF lengths of 487.20–538.24 bp. Across the complete 18-sample sequencing project, 4,264,940 genes were predicted before dereplication, and clustering generated a non-redundant catalogue of 2,584,135 genes with a combined length of approximately 1.49 Gb. Of these catalogue genes, 2,394,724 (92.67%) received NR annotations, 1,770,238 (68.50%) KEGG annotations, and 2,009,511 (77.76%) COG annotations. Detailed per-sample sequencing, assembly, and accession information, including N90, maximum contig length, and mean ORF length, is provided in Supplementary Table S1. These sequencing, assembly, and annotation steps used established tools and databases, including fastp, MEGAHIT, Prodigal, CD-HIT, DIAMOND, LEfSe, KEGG, and COG [25,26,27,28,29,30,31,32].

### 3.2. Alpha and Beta Diversity of the Chundzha and Rakhman Microbial Communities

Alpha-diversity analysis showed a directional difference in species richness between sites. Mean Sobs/ACE/Chao1 richness was 27,417 ± 67 in Chundzha and 20,012.7 ± 466.2 in Rakhman. The between-site richness difference did not reach statistical significance after multiple-testing correction (two-tailed Wilcoxon rank-sum test, *p* = 0.0809; FDR-adjusted *q* = 0.1078). The identical Sobs, ACE, and Chao1 values within each sample resulted from the absence of singleton species in the species-level read-number profiles under the Majorbio estimator implementation, rather than from a user-defined rare-taxon filtering threshold. Individual sample-level alpha-diversity values are provided in Supplementary Table S2.

Shannon diversity was similar between sites, with mean values of 7.081 ± 0.139 for Chundzha and 7.228 ± 0.265 for Rakhman (*p* = 0.6625; *q* = 0.6625). Thus, the data support a directional richness pattern but not a statistically confirmed between-site difference in alpha diversity.

Genus-level Bray–Curtis ordination showed apparent separation of the Chundzha and Rakhman samples. In the PCoA, PC1 explained 87.63% of the variation and PC2 explained 6.83%. Chundzha replicates formed a compact cluster, whereas Rakhman samples showed greater spread along PC2 (Figure 2C).

PERMANOVA [34] attributed 87.30% of the variation in genus-level community composition to sampling site (pseudo-*F* = 27.48, *R*^2^ = 0.873), but the permutation-derived *p*-value was 0.10 and therefore did not provide statistical support at *p* < 0.05. ANOSIM [33] similarly yielded *R* = 1.000 with *p* = 0.093 (999 permutations).

Homogeneity-of-dispersion testing using PERMDISP [35] did not detect a statistically significant difference in within-group dispersion between Chundzha and Rakhman (PERMDISP, *F* = 5.278, permutation *p* = 0.10). Mean distances to the group centroid were 0.109 for Chundzha and 0.160 for Rakhman. Thus, the visually greater dispersion of the Rakhman samples was not statistically supported. The multivariate statistical summary is provided in Supplementary Table S3, and the underlying Bray–Curtis distance matrix and PERMDISP distances are provided in Supplementary Table S9.

NMDS based on the same Bray–Curtis dissimilarity matrix showed a similar apparent ordination pattern, with a platform-reported stress value of 0.000 (Figure 2D). Given the six-sample dataset and the non-significant permutation tests, this near-perfect two-dimensional fit was interpreted cautiously and was not considered independent evidence of statistically significant community differentiation.

### 3.3. Taxonomic Composition and Community Structure

Taxonomic profiling revealed reproducible site-associated patterns in relative community composition (Figure 3A,B), while the limited replication precluded treating these patterns as statistically established community differences. At the genus level, Chundzha showed comparatively higher representation of *Salegentibacter*, *Halomonas*, *Halalkalicoccus*, *Salinimicrobium*, *Kocuria*, *Nocardioides*, and *Marinobacter*, whereas Rakhman showed higher relative representation of *Bradyrhizobium*, *Sphingomonas*, *Pseudolabrys*, *Rugositalaea*, and Candidatus *Udaeobacter*. The genus-level heatmap showed a corresponding site-associated pattern across the three spatial replicates (Figure 3C). The processed genus-level NR RPKM abundance matrix is provided in Supplementary Table S8.

Bacteria dominated the taxonomically assigned reads at both sites, but Archaea contributed a larger pooled proportion in Chundzha (approximately 9.4%) than in Rakhman (approximately 2.2%). The Chundzha archaeal fraction included prominent Methanobacteriota/Halobacteria-related lineages such as *Halalkalicoccus*, *Natrinema*, and *Natronococcus*, whereas Rakhman showed comparatively stronger representation of Candidatus Bathyarchaeota/Bathyarchaeia-related lineages. Sample- and site-level Bacteria/Archaea proportions are provided in Supplementary Table S7.

At broader taxonomic levels, the two communities shared many bacterial and archaeal lineages but differed in their relative contributions. These taxonomic profiles are therefore best interpreted as site-associated compositional patterns rather than complete replacement of high-level lineages. Taxonomic affiliation alone was not used to assign metabolic functions to individual genera; functional results were interpreted independently at the metagenome level. Taxonomic assignments were generated from DIAMOND-NR best-hit annotation, and exploratory differential features were evaluated using LEfSe [29,30].

### 3.4. Differential Taxonomic Signatures Between Chundzha and Rakhman

Exploratory LEfSe analysis highlighted genus-level features contributing to the apparent between-site taxonomic contrast. Among the strongest Chundzha-associated genera were *Nocardioides* (LDA = 4.166), *Salegentibacter* (LDA = 4.510), *Salinimicrobium* (LDA = 4.457), and *Halomonas* (LDA = 4.433). Rakhman-associated genus-level features included *Bradyrhizobium* (LDA = 4.832) and Candidatus *Udaeobacter* (LDA = 4.483) (Figure 4).

All genera shown in the principal LEfSe figure met the nominal criterion of *p* < 0.05 and LDA > 4.0. Because no multiple-testing correction was applied within the LEfSe feature set and only three spatial replicates were available per site, these taxa are presented as exploratory discriminant features rather than validated biomarkers.

The complete multi-rank LEfSe output is provided in Supplementary Table S4, and the phylogenetic cladogram has been moved to Supplementary Figure S1 to reduce redundancy in the main text.

The exploratory LEfSe features were broadly consistent with the relative-abundance profiles and hierarchical clustering; however, this concordance was interpreted descriptively rather than as independent statistical validation.

### 3.5. Functional Profiles of the Chundzha and Rakhman Metagenomes

Functional annotation showed that both geothermal communities contained a broad repertoire of genes involved in core cellular processes and metabolism. The most represented COG categories included amino acid transport and metabolism, general function prediction, energy production and conversion, carbohydrate transport and metabolism, lipid transport and metabolism, translation and ribosomal biogenesis, cell wall/membrane/envelope biogenesis, signal transduction, and inorganic ion transport and metabolism (Figure 5A).

When COG functions were summarized into broader Level 1 categories, both Chundzha and Rakhman were dominated by genes assigned to Metabolism, followed by Cellular Processes and Signaling and Information Storage and Processing. The two sites showed directional differences in these broad functional categories, but none of the Level 1 comparisons reached the conventional significance threshold in the two-group Wilcoxon analysis (*p* = 0.0809 for the major categories examined). Accordingly, these differences were treated as trends in relative functional representation rather than statistically supported between-site differences.

KEGG pathway analysis provided a more detailed view of these functional patterns. At KEGG Pathway Level 2, Chundzha showed higher relative representation of functions assigned to carbohydrate metabolism, amino acid metabolism, metabolism of cofactors and vitamins, lipid metabolism, and global and overview maps (Figure 5B). Rakhman, in contrast, showed higher relative representation of energy metabolism, signal transduction, cellular community–prokaryotes, and membrane transport. The differences followed a consistent direction across the three spatial replicates within each site, but the individual two-group comparisons yielded *p* = 0.0809 and were therefore not considered statistically significant.

At KEGG Pathway Level 3, Chundzha showed comparatively higher representation of broad biosynthetic functions, including metabolic pathways, biosynthesis of secondary metabolites, biosynthesis of cofactors, and biosynthesis of amino acids (Figure 5C). Rakhman showed higher relative representation of microbial metabolism in diverse environments, two-component systems, carbon metabolism, ABC transporters, quorum sensing, and purine metabolism. As observed at Level 2, these pathway-level contrasts represented directional differences rather than statistically significant effects under the present sample size.

The broad functional profiles were largely shared between sites, with directional differences in the relative representation of selected pathway categories. Chundzha tended to show greater representation of several biosynthetic and nutrient-metabolism functions, whereas Rakhman showed relatively greater representation of selected energy-metabolism, signaling, and transport functions; these contrasts were not statistically significant under the present sample size. The KEGG Level 2 and Level 3 comparison is summarized in Supplementary Table S5.

### 3.6. Targeted Metabolic Potential Related to Hydrogen, Nitrogen, Carbon, Lipid, and Fermentative Metabolism

To examine candidate functions of particular relevance to energy metabolism and biotechnology, TPM-normalized KEGG Orthology groups associated with hydrogen metabolism, nitrogen fixation, RuBisCO-associated carbon fixation, lipid metabolism, and fermentation were compared between sites (Figure 6).

Several hydrogenase-associated KOs showed higher mean relative representation in the Rakhman metagenomes. Uptake hydrogenase genes *hupL* and *hupS* and maturation genes *hypA*, *hypB*, *hypD*, and *hypF* showed directional differences, but none remained statistically significant after FDR correction. These annotations indicate hydrogenase-related genomic potential and do not establish hydrogenase class diversity or net hydrogen-production capacity (Figure 6A).

The KO initially selected as *hydA* (K00533) was not detected in the targeted table. Because *hydA* is used for different hydrogenase classes and is not represented by a single universal KO, this observation was not interpreted as evidence for the absence of hydrogen-producing hydrogenases.

Nitrogenase-associated genes, including *nifD*, *nifH*, *nifE*, *nifK*, and *nifN*, showed higher mean representation in Rakhman. None of these targeted differences remained significant after FDR correction; accordingly, they are interpreted as candidate differences in nitrogen-fixation-associated genomic potential rather than evidence of active nitrogen fixation (Figure 6B).

RuBisCO-associated genes showed a similar directional pattern. *rbcL* and *rbcS* were higher on average in Rakhman, but the comparisons were not statistically significant after FDR correction. These markers represent Calvin–Benson–Bassham-cycle genomic potential only and do not establish active carbon fixation or capture alternative autotrophic carbon-fixation pathways (Figure 6B).

Lipid-metabolism genes showed a mixed distribution: *accA*, *accC*, *accD*, *fabF*, *fabI*, and *plsB* were relatively higher in Rakhman, whereas *fabD*, *fabG*, and *plsC* were higher in Chundzha. The acyltransferase-associated ortholog K00635 was nearly identical between sites (approximately 0.0480% in Chundzha and 0.0466% in Rakhman; *p* = 1.0) (Figure 6C).

Fermentation-related genes were detected in both communities. Chundzha showed moderately higher mean representation of *hbd*, *crt*, and *bcd*, whereas *adhE* was somewhat higher in Rakhman; thl/*atoB* and *etfA*/*etfB* were broadly comparable. These patterns indicate genomic potential for fermentative functions but do not demonstrate a complete or active solventogenic pathway (Figure 6C).

Overall, hydrogenase-, nitrogenase-, and RuBisCO-associated genes showed the strongest directional contrasts, with higher mean representation in Rakhman. Lipid- and fermentation-associated genes showed mixed distributions. Photosynthesis-associated KOs occurred at low abundance, and none remained significant after FDR correction; these findings are therefore considered secondary exploratory observations. Complete targeted KO abundance and statistical results are provided in Supplementary Table S6.

## 4. Discussion

### 4.1. Community Structure Across Two Contrasting Geothermal-Associated Substrates

The two thermally influenced substrate communities showed pronounced but statistically unconfirmed compositional contrasts. Chundzha exhibited a directional trend toward higher species richness, whereas Shannon diversity was similar between sites. Genus-level PCoA and NMDS showed apparent separation, but PERMANOVA (R^2^ = 0.873, *p* = 0.10), ANOSIM (R = 1.000, *p* = 0.093), and PERMDISP (F = 5.278, *p* = 0.10) did not provide conventional statistical support under the present replication. These effect sizes and ordination patterns are therefore interpreted as exploratory evidence of site-associated structuring rather than proof of discrete community states.

The ecological context is nevertheless informative. The analyzed matrix was not surrounding ambient soil: it was moist substrate in direct contact with thermal water. Chundzha waters measured approximately 38–50 °C and pH 7–8 during sampling, whereas Rakhman waters were approximately 30–40 °C with pH about 8. These moderate thermal regimes, combined with the substrate–water interface, make it plausible that the recovered communities integrate both geothermal selection and microbial input from the surrounding terrestrial environment.

Such mixed community signatures are consistent with the heterogeneity reported across terrestrial geothermal systems. Hot-spring microbiomes vary with temperature, pH, mineralization, hydrochemistry, and sampled matrix, and moderate-temperature substrates are not necessarily dominated by canonical thermophiles [3,4,5,6,7,8,9,10,37,38,39,40,41,42,43]. In Kazakhstan, the Zharkent geothermal spring was predominantly bacterial [21], whereas genome-resolved work in other geothermal systems has revealed substantial archaeal diversity [12,15,44,45]. These comparisons support interpreting Chundzha and Rakhman as geothermal-associated ecological interfaces rather than exclusively thermophilic assemblages. Recent large-scale and regional surveys likewise emphasize substantial among-spring heterogeneity and the value of paired physicochemical characterization when interpreting community structure [13,14].

The contrasting taxonomic profiles are ecologically compatible with the broader settings of the two systems. Chundzha contained prominent halophilic and halotolerant genera, consistent with an arid basin and mineralized groundwater context, whereas Rakhman contained more soil-associated Alphaproteobacteria and Acidobacteriota/Verrucomicrobiota-related lineages typical of a cooler mountainous terrestrial interface. These links remain hypotheses because matched replicate-level measurements of salinity, conductivity, major ions, and other geochemical variables were not available and were not used as statistical explanatory covariates.

Archaeal composition provided an additional contrast. Archaea represented a larger pooled fraction of taxonomically assigned reads in Chundzha than in Rakhman, with Chundzha enriched in halophilic Methanobacteriota/Halobacteria-related lineages and Rakhman showing comparatively stronger Candidatus Bathyarchaeota/Bathyarchaeia-related representation. This domain-level distinction is relevant because archaeal lineages can occupy physiologically and evolutionarily distinct niches in geothermal environments [44,45,46].

At the bacterial/genus level, the Chundzha pattern was driven by taxa such as *Halomonas*, *Salegentibacter*, *Salinimicrobium*, *Marinobacter*, *Kocuria*, and related lineages, whereas Rakhman showed higher relative representation of *Bradyrhizobium*, *Sphingomonas*, *Pseudolabrys*, *Rugositalaea*, and Candidatus *Udaeobacter*. Comparable site-to-site heterogeneity has been reported in geographically separated hot springs and hydrothermal features [3,21,41,43,47,48]. The present LEfSe features should, however, be viewed as exploratory because of the small sample size and absence of FDR correction within that analysis.

The absence of dominance by well-known hyperthermophilic genera is therefore not inconsistent with geothermal influence. The measured thermal waters were moderate rather than hyperthermal, and the sampled matrix was a substrate–water interface. The high Shannon values and occurrence of soil-associated lineages likely reflect this ecological mixing rather than demonstrating that the samples were unrelated to the geothermal systems.

The clustering of the three spatial replicates from each site indicates reproducible compositional patterns across points separated by approximately 5 m, but with n = 3 per site it cannot establish generality across the full geothermal systems. Expanded sampling across thermal gradients and adjacent non-thermal controls will be required to partition geothermal versus surrounding terrestrial contributions.

Taxonomic affiliation alone was not used to infer specific metabolic processes. Because individual genera can contain physiologically diverse species and functional genes were not linked to metagenome-assembled genomes or taxonomically resolved contigs, the taxonomic and functional patterns are interpreted as parallel properties of the community metagenomes rather than direct gene-to-taxon associations.

### 4.2. Shared Functional Core and Site-Specific Functional Patterns

Despite the taxonomic contrast, the Chundzha and Rakhman metagenomes shared a broad functional repertoire. COG and KEGG annotations showed abundant functions related to amino acid and carbohydrate metabolism, energy production, lipid metabolism, membrane processes, replication, translation, and environmental signaling at both sites. Similar broad metabolic repertoires, including pathways related to carbohydrate, amino acid, and energy metabolism, have been reported in geographically distinct geothermal systems [43,49,50]. Recent hybrid metagenomic analyses of hot-spring biofilms further demonstrate the value of MAG reconstruction for linking community diversity with carbohydrate degradation, stress adaptation, and biofilm-associated traits [51].

The functional contrasts were less pronounced than the taxonomic patterns. Most broad COG and KEGG comparisons were not statistically significant, and the data therefore support a largely shared functional framework with directional shifts in selected categories. This pattern is consistent with the possibility of community-level functional redundancy, but the present gene-catalogue analysis does not directly quantify redundancy among individual taxa or genomes [52,53].

Chundzha showed comparatively greater representation of pathways related to carbohydrate and amino acid metabolism, cofactors and vitamins, lipid metabolism, and several biosynthetic categories. In contrast, Rakhman showed greater representation of energy metabolism, membrane transport, signal transduction, two-component systems, and microbial metabolism in diverse environments. Similar site-associated shifts in broad metabolic categories have been reported in comparative geothermal metagenomic studies [43,49].

The latter pattern may indicate a relatively greater genomic investment in environmental sensing, substrate acquisition, and energy-related functions in the Rakhman community. However, these pathway categories are broad and contain genes participating in multiple physiological processes. They should therefore not be interpreted as direct evidence of higher metabolic activity or environmental stress at Rakhman.

The preservation of similar broad functional categories alongside contrasting taxonomic profiles is compatible with functional overlap among different community members [54]. However, this remains an inference: demonstrating functional redundancy would require genome-resolved assignment and quantitative comparison of functional repertoires among taxa or MAGs.

In contrast to the relatively consistent Rakhman-associated patterns for hydrogen, nitrogen fixation, and carbon fixation, lipid- and fermentation-related genes showed mixed distributions between sites. Rakhman showed higher relative representation of *accA*, *accC*, *accD*, *fabF*, *fabI*, and *plsB*, whereas *fabD*, *fabG*, and *plsC* were higher in Chundzha. Chundzha also showed moderately higher representation of *hbd*, *crt*, and *bcd*, while *adhE* was somewhat higher in Rakhman. These genes participate in central cellular metabolism and multiple pathway contexts; therefore, their relative abundance does not by itself indicate enhanced lipid accumulation or a complete solventogenic pathway [55].

Photosynthesis-associated KOs were detected only at low relative abundance and showed no FDR-significant between-site differences. Although several photosystem and phycocyanin-associated genes showed higher mean representation in Chundzha, this pattern is best treated as a secondary feature. Cyanobacterial communities have previously been reported from thermal springs in the Almaty region [22], and photosynthetic populations can contribute distinct functional repertoires in geothermal microbial mats [53], but the present data do not support differential photosynthetic activity between the two sites.

### 4.3. Hydrogen Metabolism and Hydrogenase Maturation Potential

Several of the largest directional differences in the targeted KO panel involved hydrogenase-associated genes. Uptake hydrogenase structural genes *hupL* and *hupS* and maturation genes *hypA*, *hypB*, *hypD*, and *hypF* showed higher mean relative representation in Rakhman, but none of these comparisons remained significant after FDR correction.

The co-occurrence of structural hydrogenase genes and multiple hyp maturation genes is biologically consistent with hydrogenase-related genomic machinery because functional [NiFe]-hydrogenases require accessory proteins for assembly of the catalytic metal center [56,57]. Nevertheless, KO-level annotation alone is insufficient to resolve hydrogenase class diversity or infer net hydrogen production.

Hydrogen metabolism is widespread among microorganisms inhabiting geothermal and subsurface environments, where molecular hydrogen can serve as an electron donor or metabolic intermediate. Genomic and metagenomic surveys show that hydrogenases are distributed across diverse microbial lineages and ecosystems [58]. Geothermal metagenomes likewise contain multiple hydrogenase classes associated with energy conservation under chemically reduced conditions [1,15]. Because hydrogenases differ in physiological function, the presence of *hupSL* is most consistent with uptake-hydrogenase potential and should not be interpreted as evidence for net biological hydrogen production [58].

An additional caution concerns the targeted search for *hydA*. The absence of K00533 from the selected KO table cannot be equated with the absence of hydrogen-evolving hydrogenases because hydrogenases comprise phylogenetically and functionally distinct groups that are not represented by a single universal KO identifier [58]. A dedicated hydrogenase classification based on catalytic-subunit phylogeny or specialized databases would be required to distinguish hydrogen-producing and hydrogen-consuming hydrogenases with greater confidence.

Thus, the present data identify a candidate Rakhman-associated trend in hydrogenase-related genomic potential, particularly for uptake hydrogenase and maturation functions, but do not establish the direction, rate, or activity of hydrogen metabolism in situ.

### 4.4. Nitrogen and Carbon Fixation Potential and Biotechnological Implications

Nitrogenase-associated genes showed some of the largest directional fold differences in the targeted panel. *nifH*, *nifD*, *nifK*, *nifE*, and *nifN* were higher on average in Rakhman; however, none of these comparisons remained significant after FDR correction.

The simultaneous detection of multiple components of the nitrogenase system is more consistent with genomic nitrogen-fixation potential than detection of *nifH* alone. Biological nitrogen fixation has been demonstrated experimentally in high-temperature geothermal communities using acetylene-reduction- and isotope-based approaches [16,59].

Nevertheless, the present comparison does not demonstrate active nitrogen fixation. First, most targeted between-site comparisons did not meet the statistical significance threshold after correction. Second, metagenomic data establish gene presence and relative representation but do not determine whether the genes were transcriptionally active at the time of sampling. Confirmation would require metatranscriptomic analysis or direct nitrogenase activity measurements, as used in previous geothermal studies [16,59].

RuBisCO-associated genes showed a similar directional pattern. *rbcL* and *rbcS*, representing the Calvin–Benson–Bassham cycle, were higher on average in Rakhman. These markers were selected as targeted indicators rather than comprehensive coverage of microbial carbon fixation; alternative pathways such as the reverse TCA, Wood–Ljungdahl, 3-hydroxypropionate, and dicarboxylate/4-hydroxybutyrate pathways were not systematically evaluated [60].

The parallel representation of nitrogenase-related genes, RuBisCO-associated genes, and hydrogenase-related functions in Rakhman is particularly notable. These functions should not be assumed to occur within the same organisms without genome-resolved linkage. Recent geothermal genome studies have shown that carbon, nitrogen, and energy-metabolism traits can co-occur within individual lineages, but such linkage must be demonstrated at the genome level [45,61]. Recent large-scale genome-resolved analysis of terrestrial geothermal springs further showed that archaeal community structure and metabolic potential, including carbon, nitrogen, sulfur, and hydrogen metabolism, vary substantially across physicochemical gradients [45].

This combination provides a useful basis for subsequent genome-resolved metagenomic or metatranscriptomic analysis aimed at determining whether these functions are taxonomically linked and whether they are expressed under in situ conditions.

These targeted functional patterns are also relevant from a biotechnological perspective. Their principal value lies in identifying candidate functions for further investigation rather than in demonstrating biotechnology-ready phenotypes. Geothermal microbiomes are recognized as reservoirs of thermostable enzymes and diverse metabolic systems [42,43]. The Rakhman-associated combination of hydrogenase maturation, nitrogenase, and RuBisCO-related genes represents a priority for genome-resolved and expression-based follow-up, whereas the Chundzha-associated fermentative and photosynthesis-related signatures provide complementary targets. Regional evidence from the high-altitude Tamdykul geothermal spring in Tajikistan likewise demonstrates the value of genome-resolved metagenomics for identifying thermophilic archaeal lineages and hydrogenase-containing genomes in Central Asian geothermal environments [15].

### 4.5. Limitations and Future Directions

Several limitations should be considered when interpreting these results. First, only three spatial replicates were analyzed per site, limiting statistical power and generalization across each geothermal system. Second, matched physicochemical measurements were not available for every metagenomic replicate; thermal-water temperature and pH were recorded during sampling, but salinity, conductivity, major ions, and other geochemical variables were unavailable at equivalent replicate resolution. Consequently, proposed environmental associations remain ecological hypotheses rather than statistically demonstrated drivers. Third, shotgun metagenomics describes genomic potential rather than gene expression or metabolic flux. Fourth, the functional analysis was gene-catalogue-based and did not include MAG reconstruction or contig-resolved gene-to-taxon linkage, preventing assignment of the detected functions to specific microbial lineages. Finally, the broad functional similarity is consistent with, but does not directly demonstrate, community-level functional redundancy, and the targeted carbon-fixation panel focused on RuBisCO-associated markers rather than all autotrophic pathways.

Future work should therefore combine expanded spatial and temporal sampling with matched hydrochemical characterization, metagenome-assembled genome (MAG) reconstruction, metatranscriptomic analysis, and targeted experimental assays. Genome-resolved approaches would help determine which organisms carry the hydrogenase-, nitrogenase-, carbon-fixation, fermentation-, lipid-, and photosynthesis-associated functions detected here, while expression-based and physiological measurements would be needed to establish whether these pathways are active under in situ conditions [12,18]. Hybrid short- and long-read metagenomic approaches have also improved MAG recovery and functional linkage in hot-spring biofilms [51].

## 5. Conclusions

This comparative shotgun metagenomic study characterized microbial communities associated with thermally influenced substrates from the Chundzha and Rakhman geothermal systems in Kazakhstan. The two sites showed pronounced site-associated taxonomic patterns, including apparent genus-level ordination separation, although the between-site effect was not statistically supported by permutation-based testing under the present replication. Chundzha showed a directional trend toward higher species richness, whereas Shannon diversity was similar between sites.

Despite the taxonomic contrast, broad functional profiles were largely shared. Several hydrogenase-, nitrogenase-, RuBisCO-, lipid-metabolism-, fermentation-, and photosynthesis-associated genes showed directional differences in relative representation, but none of the targeted KO comparisons remained significant after FDR correction. These findings therefore identify candidate taxonomic and functional patterns rather than statistically validated site-specific signatures. The results provide a baseline resource for geothermal-associated microbial communities in Kazakhstan; future studies incorporating larger sample sizes, matched geochemical measurements, metatranscriptomics, and genome-resolved reconstruction will be required to determine environmental drivers, taxonomic carriers, and activity of the metabolic functions highlighted here.

## Figures and Tables

**Figure 1:**
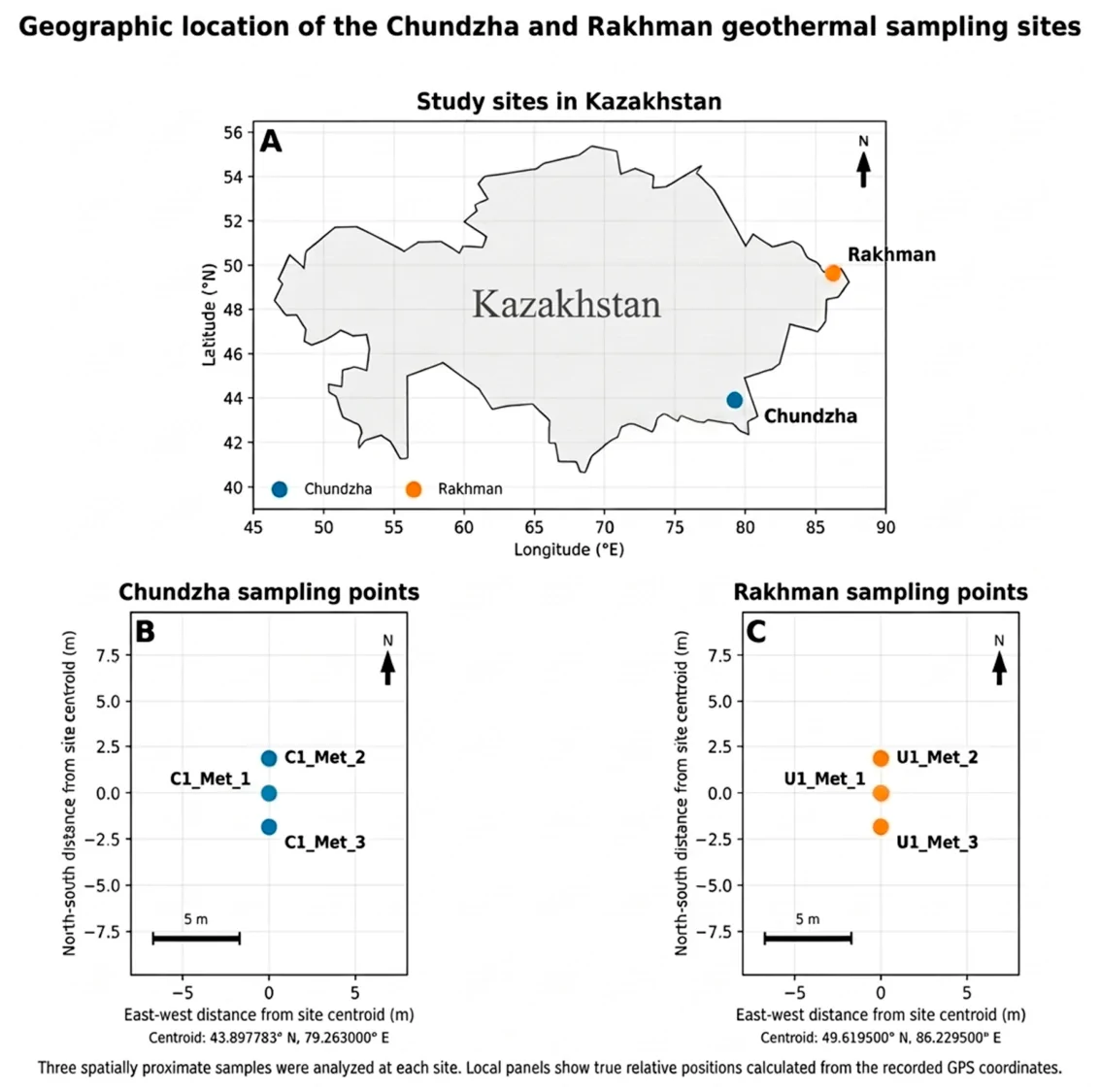
Geographic location of the Chundzha and Rakhman geothermal sampling sites in Kazakhstan. (**A**) Locations of the Chundzha and Rakhman geothermal systems within Kazakhstan. (**B**) Relative spatial positions of three sampling points at the Chundzha site. (**C**) Relative spatial positions of three sampling points at the Rakhman site. Three independently collected spatial replicates were analyzed per site; replicate points were approximately 5 m apart, and the sampled moist substrate was directly exposed to thermal water.

**Figure 2:**
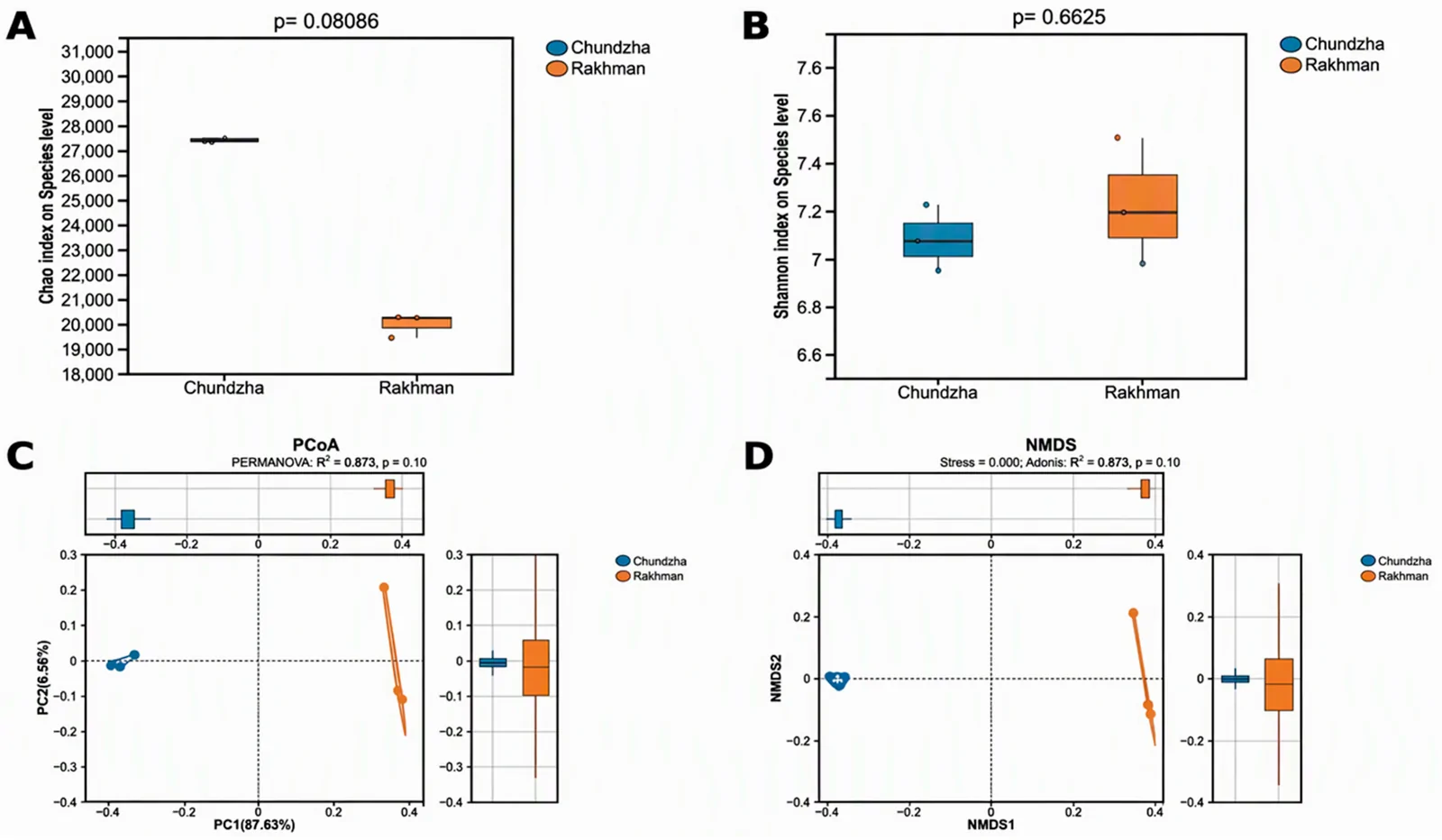
Alpha- and beta-diversity patterns of microbial communities from Chundzha and Rakhman. (**A**) Species-level Chao1 richness and (**B**) Shannon diversity calculated from NR-based read-number abundance profiles. Group differences were evaluated using two-tailed Wilcoxon rank-sum tests with FDR correction. (**C**) PCoA based on genus-level RPKM-normalized Bray–Curtis dissimilarities (PC1 = 87.63%, PC2 = 6.83%; PERMANOVA *R*^2^ = 0.873, *p* = 0.10). (**D**) NMDS based on the same distance matrix (platform-reported stress = 0.000; Adonis *R*^2^ = 0.873, *p* = 0.10). ANOSIM yielded *R* = 1.000, *p* = 0.093. PERMDISP showed no statistically significant difference in dispersion (*F* = 5.278, *p* = 0.10). Neither permutation-based between-site test reached *p* < 0.05.

**Figure 3:**
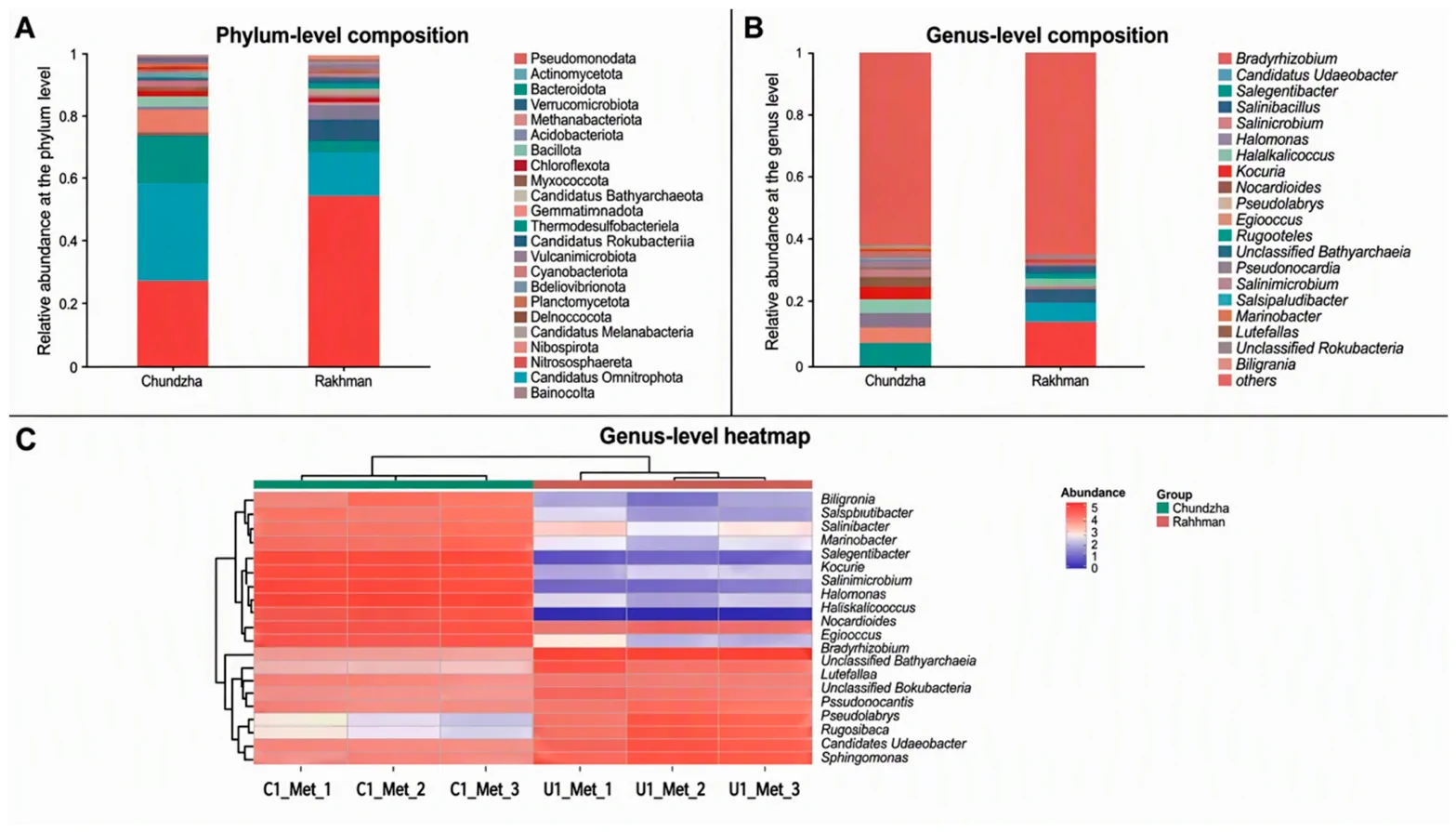
Taxonomic composition of microbial communities associated with the Chundzha and Rakhman geothermal systems based on NCBI NR-derived assignments. (**A**) Relative abundance of dominant phyla and (**B**) dominant genera using RPKM-normalized abundance profiles. Lower-abundance taxa not included among the displayed top-ranked taxa were grouped as “Others” for visualization only and were not removed before alpha-diversity analysis. (**C**) Hierarchical heatmap of genus-level profiles across the six spatial replicates.

**Figure 4:**
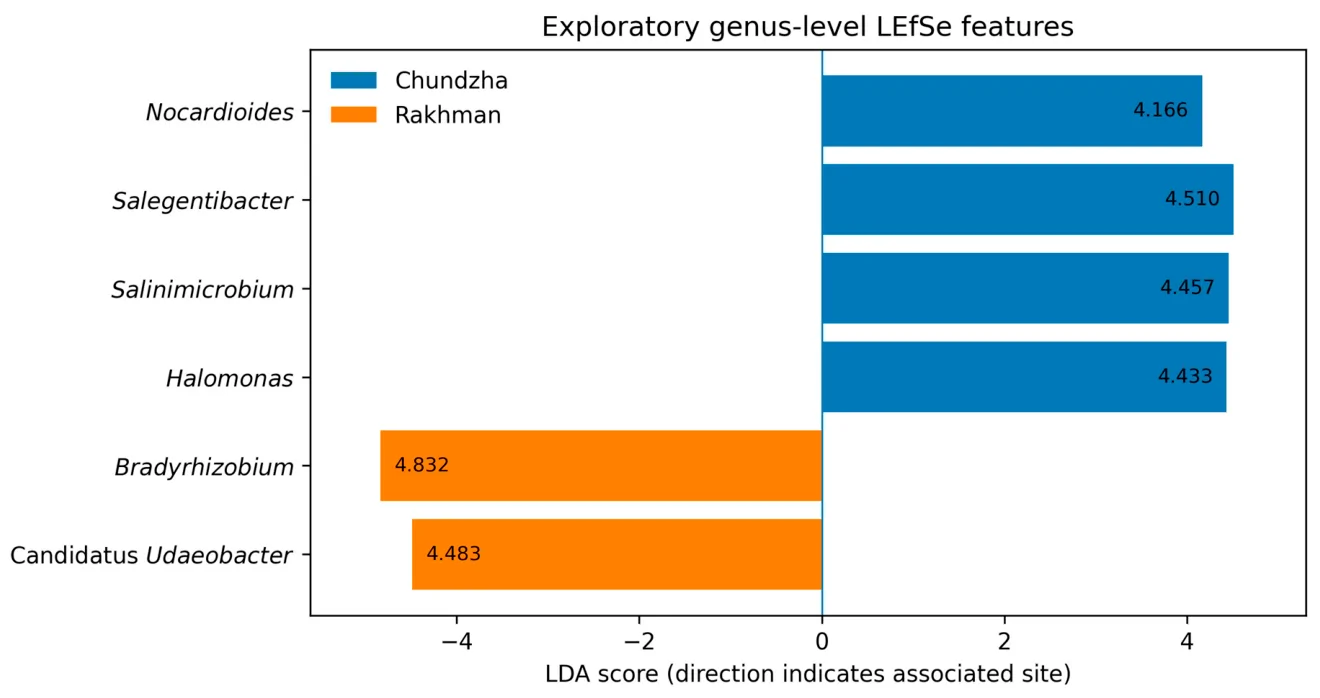
Exploratory genus-level LEfSe features associated with Chundzha and Rakhman. Only genera meeting the nominal LEfSe criterion of *p* < 0.05 and LDA > 4.0 are displayed. No multiple-testing correction was applied within the LEfSe feature set; therefore, these taxa should be interpreted as exploratory discriminant features rather than validated biomarkers. Positive bars indicate Chundzha-associated features and negative bars indicate Rakhman-associated features; labels report absolute LDA scores.

**Figure 5:**
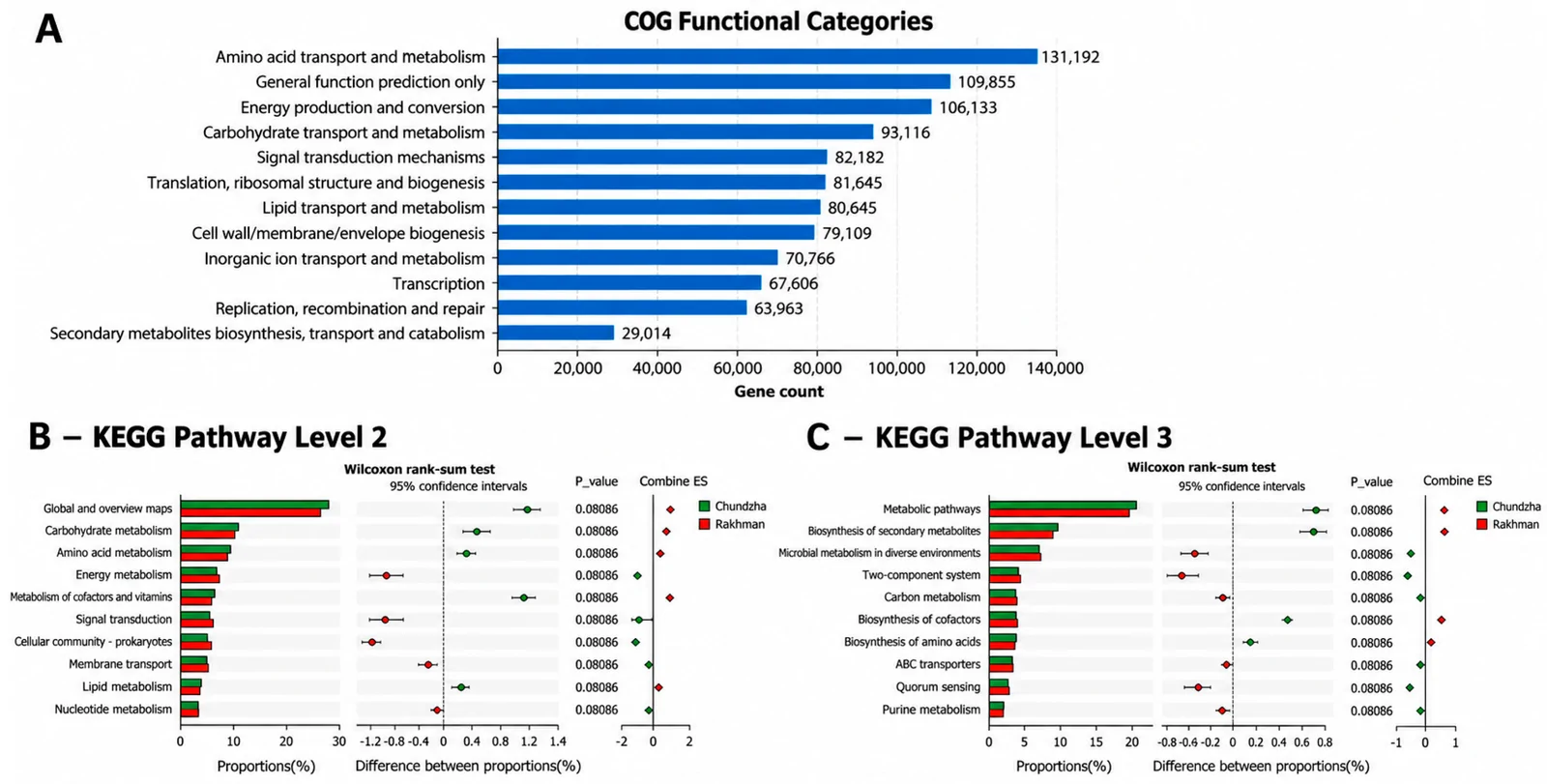
Broad functional profiles of the Chundzha and Rakhman metagenomes. (**A**) Distribution of major COG functional categories. (**B**) Selected KEGG Pathway Level 2 categories and (**C**) selected KEGG Pathway Level 3 pathways. Bars represent mean abundance across three spatial replicates; error bars indicate standard deviation (SD). Between-site comparisons used two-tailed Wilcoxon rank-sum tests. The displayed differences are interpreted as directional trends unless statistically supported. Nominal two-tailed Wilcoxon *p*-values are reported for these selected broad COG/KEGG comparisons; no additional FDR adjustment was applied to this exploratory broad-category panel.

**Figure 6:**
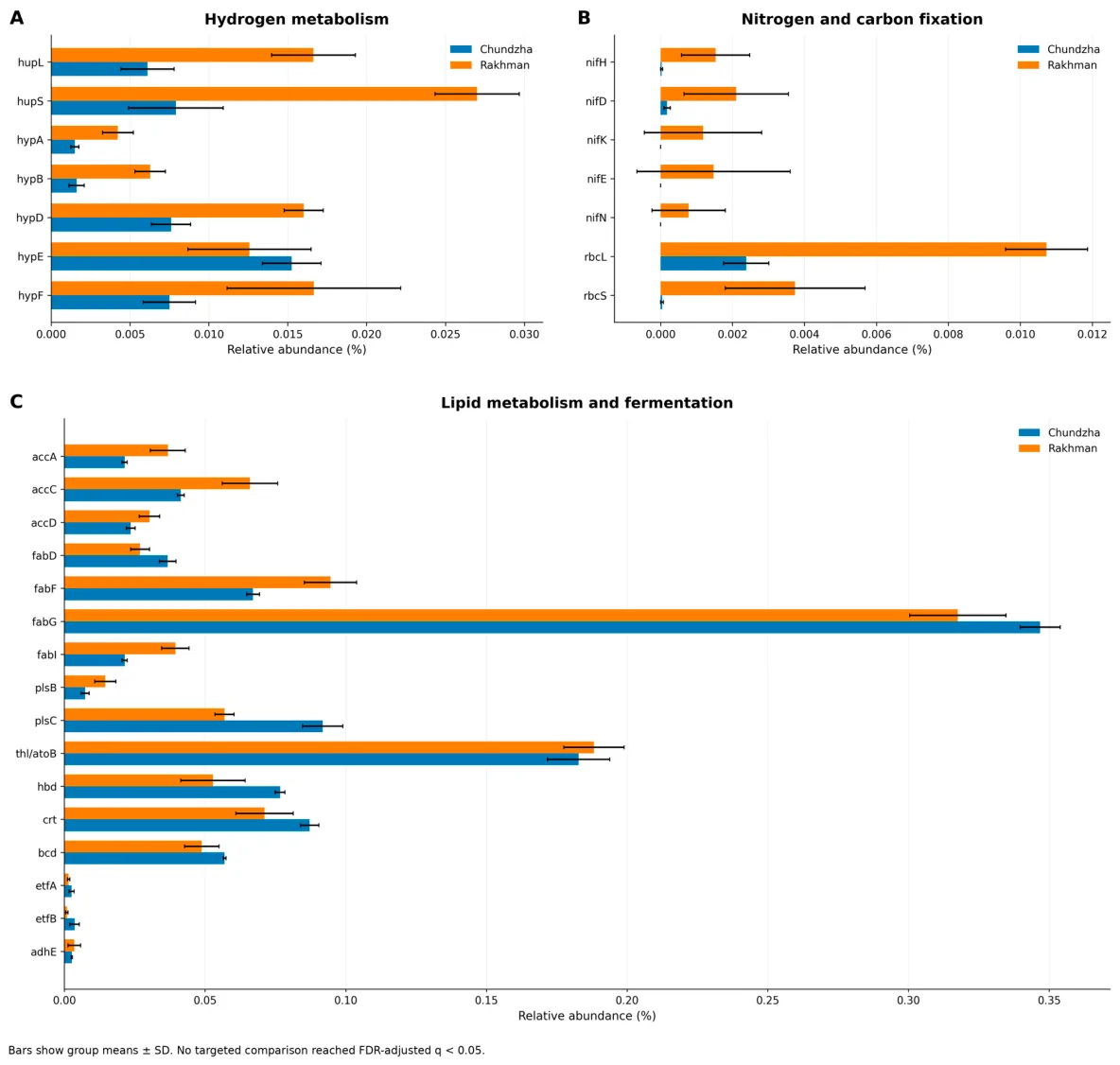
Targeted comparison of selected metabolic functions in the Chundzha and Rakhman metagenomes using TPM-normalized KO abundances. (**A**) Hydrogen uptake and hydrogenase maturation; (**B**) nitrogen fixation and RuBisCO-associated carbon fixation; (**C**) lipid metabolism and fermentation. Bars show group means across three spatial replicates and error bars indicate SD. Nominal *p*-values were calculated using two-tailed Wilcoxon rank-sum tests and adjusted across the complete targeted KO set using FDR correction. None of the displayed comparisons reached q < 0.05; therefore, the differences are interpreted as directional candidate trends rather than statistically supported enrichment.

**Table 1:** Geographic characteristics and sample codes of the Chundzha and Rakhman geothermal sampling sites.

Sample Type	Elevation (m)	Longitude (E)	Latitude (N)	Sample Code	Site
Moist substrate directly exposed to thermal water	700	79°15.783′ E	43°53.867′ N	C1_Met_1	Chundzha
	700	79°15.783′ E	43°53.868′ N	C1_Met_2	Chundzha
	700	79°15.783′ E	43°53.866′ N	C1_Met_3	Chundzha
Moist substrate directly exposed to thermal water	1750	86°13.770′ E	49°37.170′ N	U1_Met_1	Rakhman
	1750	86°13.770′ E	49°37.171′ N	U1_Met_2	Rakhman
	1750	86°13.770′ E	49°37.169′ N	U1_Met_3	Rakhman

**Table 2:** Sequencing, quality-control, assembly, and gene-prediction characteristics of the six metagenomic samples.

Predicted ORFs	N50 (bp)	Assembly Length (Mb)	Contigs	Reads Retained (%)	Clean Data (Gb)	Clean Reads	Raw Data (Gb)	Raw Reads	Sample ID	Site
235,298	808	138.144	159,039	99.48	6.528	43,322,506	6.576	43,548,918	C1_Met_1	Chundzha
285,486	855	172.270	189,848	99.46	6.717	44,579,030	6.768	44,819,966	C1_Met_2	Chundzha
322,892	854	191.941	213,037	99.48	6.716	44,573,580	6.766	44,807,844	C1_Met_3	Chundzha
120,516	680	65.548	91,171	99.49	6.634	44,025,572	6.682	44,252,740	U1_Met_1	Rakhman
170,501	715	95.294	127,190	99.40	7.292	48,405,542	7.354	48,699,630	U1_Met_2	Rakhman
144,716	712	80.680	107,948	99.46	6.517	43,253,352	6.567	43,489,880	U1_Met_3	Rakhman

## Data Availability

The raw shotgun metagenomic sequencing data generated in this study are available through the NCBI Sequence Read Archive under BioProject PRJNA1508369. The six samples correspond to BioSample accessions SAMN62231879, SAMN62231880, SAMN62231881, SAMN62231885, SAMN62231886, and SAMN62231887, with SRA accessions SRR40083285, SRR40083284, SRR40083276, SRR40083272, SRR40083271, and SRR40083270, respectively. Processed datasets supporting the analyses, together with additional documentation of the field sampling and sample-handling procedure, are provided in the Supplementary Materials. These include taxonomic abundance tables, alpha-diversity results, exploratory LEfSe output, targeted KO abundance/statistical tables, Bacteria/Archaea proportions, beta-diversity/statistical outputs, and Supplementary Method S1.

## Supplementary Materials

The following supporting information can be downloaded at https://www.mdpi.com/article/10.3390/biology15191704/s1, Supplementary Method S1. Field Sampling and Sample Handling Protocol for Shotgun Metagenomic Analysis; Table S1. Sampling, sequencing, assembly, and accession information for the Chundzha and Rakhman samples; Table S2. Alpha-diversity indices for individual samples; Table S3. Beta-diversity ordination and multivariate statistics; Table S4. Complete LEfSe output for taxa differentiating Chundzha and Rakhman; Table S5. KEGG Pathway Level 2 and Level 3 comparative analysis; Table S6. Targeted KEGG Orthology analysis of metabolic functions; Table S7. Relative proportions of Bacteria and Archaea in Chundzha and Rakhman metagenomes; Table S8. Processed genus-level NR RPKM abundance matrix for the six manuscript samples; Table S9. Genus-level Bray–Curtis dissimilarity matrix and PERMDISP details; Figure S1. Exploratory LEfSe cladogram.

## Abbreviations

The following abbreviations are used in this manuscript:

ACE	Abundance-based Coverage Estimator
ANOSIM	Analysis of similarities
COG	Clusters of Orthologous Groups
DNA	Deoxyribonucleic acid
FDR	False discovery rate
KEGG	Kyoto Encyclopedia of Genes and Genomes
KO	KEGG Orthology
LDA	Linear discriminant analysis
LEfSe	Linear discriminant analysis effect size
MAG	Metagenome-assembled genome
NCBI	National Center for Biotechnology Information
NMDS	Non-metric multidimensional scaling
NR	Non-redundant protein database
PCoA	Principal Coordinates Analysis
PERMANOVA	Permutational multivariate analysis of variance
RPKM	Reads per kilobase per million mapped reads
RuBisCO	Ribulose-1,5-bisphosphate carboxylase/oxygenase
Sobs	Observed species richness
TPM	Transcripts per million
