# Supplementary material for: Comparative Shotgun Metagenomics of Microbial Communities Associated with Two Geothermal Systems in Kazakhstan
Source: Biology (Basel). 2026 Sep 24;15(19):1704. doi: 10.3390/biology15191704 (PMC13663324; doi:10.3390/biology15191704)
Supplement: Supplementary file 1 [file biology-15-01704-s001.zip › Supplementary_Method_S1_Field_Sampling_Protocol.pdf]

Supplementary Method S1

Field Sampling and Sample Handling Protocol for Shotgun Metagenomic Analysis

Manuscript ID: biology-4550111

Sampling framework. Sampling procedures were adapted from the NEON Soil Biogeochemical and Microbial Sampling Protocol (NEON.DOC.014048, Revision Q) and the Earth Microbiome Project (EMP500) Sample Submission Guide, with study-specific modifications. The analyzed material consisted of moist, water-saturated soil/substrate from the shallow-water margin of the geothermal springs, where the material was in direct contact with thermal water. The samples were not collected as ambient terrestrial soil away from the springs.

Spatial sampling design. Samples were collected in July 2025 from the Chundzha thermal spring area in southeastern Kazakhstan and the Rakhman geothermal springs in eastern Kazakhstan. At each site, three spatially separated sampling points were sampled independently, providing three spatial replicates per site. Replicate points were located approximately 5 m apart.

Aseptic sampling. Samples were collected using sterile containers and sterile or appropriately decontaminated sampling tools. Sampling materials were handled to minimize cross-contamination between independent sampling points, and the three spatial replicates from each site were maintained separately during sample handling and subsequent analysis.

Table SM1. Sampling-point characteristics and field context for the six metagenomic samples.

Site	Sample ID	Latitude (N)	Longitude (E)	Elevation (m)	Sample type	Thermal-water temperature (°C)	pH	Approx. spacing
Chundzha	C1_Met_1	43°53.867′ N	79°15.783′ E	700	Moist substrate directly exposed to thermal water	38–50	7–8	~5 m
Chundzha	C1_Met_2	43°53.868′ N	79°15.783′ E	700	Moist substrate directly exposed to thermal water	38–50	7–8	~5 m
Chundzha	C1_Met_3	43°53.866′ N	79°15.783′ E	700	Moist substrate directly exposed to thermal water	38–50	7–8	~5 m
Rakhman	U1_Met_1	49°37.170′ N	86°13.770′ E	1750	Moist substrate directly exposed to thermal water	30–40	~8	~5 m
Rakhman	U1_Met_2	49°37.171′ N	86°13.770′ E	1750	Moist substrate directly exposed to thermal water	30–40	~8	~5 m
Rakhman	U1_Met_3	49°37.169′ N	86°13.770′ E	1750	Moist substrate directly exposed to thermal water	30–40	~8	~5 m

Sample collection and handling. At each sampling point, moist substrate in direct contact with thermal water was transferred directly into sterile 50 mL conical centrifuge tubes and labeled with a unique sample ID and sampling site. Samples were kept cooled during transportation at approximately 4 °C and stored at −20 °C until DNA extraction.

Environmental context. Thermal-water temperature and pH values shown above represent field measurements recorded during sampling at each geothermal site. Literature-derived hydrochemical characteristics of the two systems were used only as contextual background in the manuscript because matched replicate-level measurements of salinity, conductivity, major ions, and other geochemical variables were not available for all six samples.

Protocol references. 1. NEON. Soil Biogeochemical and Microbial Sampling Protocol (NEON.DOC.014048, Revision Q). 2. Earth Microbiome Project. EMP500 Sample Submission Guide.
