# Supplementary material for: Comparative Shotgun Metagenomics of Microbial Communities Associated with Two Geothermal Systems in Kazakhstan
Source: Biology (Basel). 2026 Sep 24;15(19):1704. doi: 10.3390/biology15191704 (PMC13663324; doi:10.3390/biology15191704)
Supplement: Supplementary file 1 [file biology-15-01704-s001.zip › Figure_S1_LEfSe.pdf]

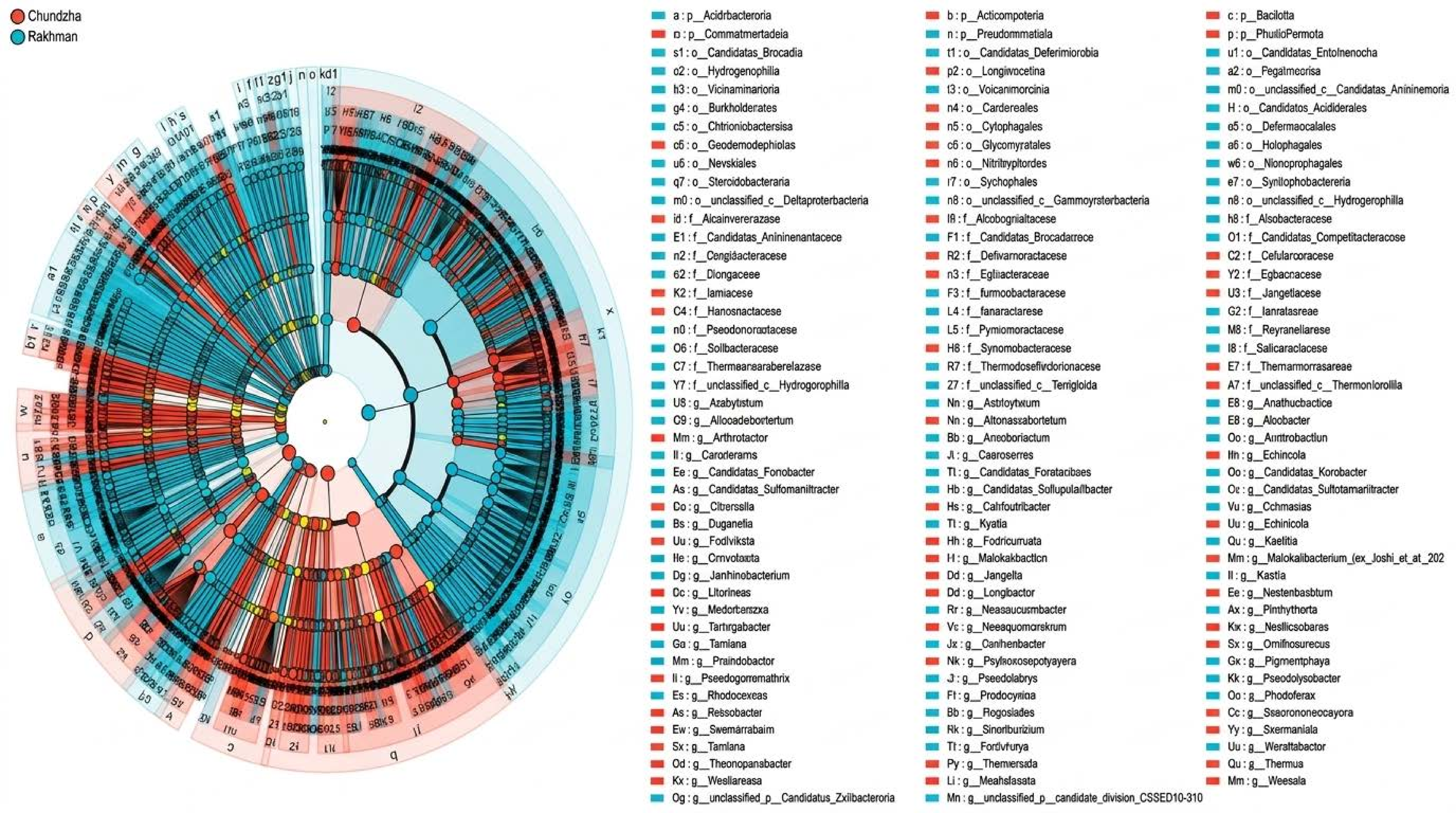
Chundzha
Rakhman
a : p__Acidrbacteroria
b : p__Acticompoteria
c : p__Bacilotta
o : p__Commatmertadeia
n : p__Preudommatiala
p : p__PhutioPermota
s1 : o__Candidatas_Brocadia
t1 : o__Candidatas_Deferimiorobia
u1 : o__Candidatas_Entolnenocha
o2 : o__Hydrogenophilia
p2 : o__Longiivocetina
a2 : o__Pegatmeorisa
h3 : o__Vicinaminarioria
t3 : o__Voicanimorcinia
m0 : o__unclassified_c__Candidatas_Anininemoria
g4 : o__Burkholderates
n4 : o__Cardereales
H : o__Candidatos_Acididerales
c5 : o__Chtrioniobactersisa
n5 : o__Cytophagales
a5 : o__Defermaocalales
c6 : o__Geodemodephiolas
c6 : o__Glycomyratales
a6 : o__Holophagales
u6 : o__Nevskiales
n6 : o__Nitritnypltordes
w6 : o__Nlonoprophagales
q7 : o__Stercidobacteraria
I7 : o__Sychophales
e7 : o__Synitophobactereria
m0 : o__unclassified_c__Deltaproterbacteria
n8 : o__unclassified_c__Gammoyrsterbacteria
n8 : o__unclassified_c__Hydrogerophilla
id : f__Alcainvererazase
I8 : f__Alcobognialtacese
h8 : f__Alsobacteracese
E1 : f__Candidatas_Anininenantacece
F1 : f__Candidatas_Brocadarrece
O1 : f__Candidatas_Competitacteracose
n2 : f__Cengidacteracese
R2 : f__Defivarnoractacese
C2 : f__Cefularcoracese
62 : f__Dlongaceee
n3 : f__Eglliacteraceae
Y2 : f__Egbacnacese
K2 : f__lamiacese
F3 : f__furmoobactaracese
U3 : f__Jangetlacese
C4 : f__Hanosnactacese
L4 : f__fanaractarese
G2 : f__Ianratasreae
n0 : f__Pseodonoraotacese
L5 : f__Pymiomoractacese
M8 : f__Reyranellarese
O6 : f__Sollbacteracese
H8 : f__Synomobacteracese
I8 : f__Salicaraclacese
C7 : f__Thermaeraraberelazase
R7 : f__Thermodosefivdorionacese
E7 : f__Thernarmorrasareae
Y7 : f__unclassified_c__Hydrogorophilla
27 : f__unclassified_c__Terrigloida
A7 : f__unclassified_c__Thermonlorollia
U8 : g__Azabytistum
Nn : g__Astrfoytxum
E8 : g__Anathucbactice
C9 : g__Allooadebortertum
Nn : g__Altonasabortetum
E8 : g__Alcobacter
Mm : g__Arthrotactor
Bb : g__Aneoboriactum
Oo : g__Anttrobactiun
Il : g__Caroderams
Jl : g__Caeroserres
Hn : g__Echincola
Ee : g__Candidatas_Fonobacter
Tl : g__Candidatas_Foratacibses
Oo : g__Candidatas_Korobacter
As : g__Candidatas_Sulfomaniltracter
Hb : g__Candidatas_Sollupulaflbacter
Oc : g__Candidatas_Sultotamarilracter
Co : g__Citerssila
Hs : g__Cahfoutribacter
Vu : g__Cchmasias
Bs : g__Duganelia
Tl : g__Kyatia
Uu : g__Echinicola
Uu : g__Fodlviksta
Hh : g__Fodricuruata
Qu : g__Kaelitia
He : g__Crnvotoxta
H : g__Malokakbacticn
Mm : g__Malokalibacterium_(ex_Joshi_et_at_202
Dg : g__Janhinobacterium
Dd : g__Jangelta
Il : g__Kastia
Dc : g__Ltorineas
Dd : g__Longibactor
Ee : g__Nestenbasbtum
Yv : g__Medorberszxa
Rr : g__Neasaucusmbacter
Ax : g__Plnthytherta
Uu : g__Tartrgabacter
Vc : g__Neeaquomorskrum
Kx : g__Nesllicsobares
Ga : g__Tamiana
Jx : g__Canhenbacter
Sx : g__Omifnosurecus
Mm : g__Praindobactor
Nk : g__Psylxoxosepotyayera
Gx : g__Pigmentphaya
Il : g__Pseedogorremathrix
J : g__Pseedolabrys
Kk : g__Pseodolysobacter
Es : g__Rhodocexeas
Ft : g__Prodocyriqa
Oo : g__Phodoferax
As : g__Rebsobacter
Bb : g__Rogosiades
Cc : g__Ssaorononeocayora
Ew : g__Swemarrabaim
Rk : g__Sinorlburizium
Yy : g__Sxermaniala
Sx : g__Tamlana
Tt : g__Fordivfurya
Uu : g__Weraftabactor
Od : g__Theonopanabacter
Py : g__Themiersada
Qu : g__Thermua
Kx : g__Wesllareasa
Li : g__Meahslasata
Mm : g__Weesala
Og : g__unclassified_p__Candidatus_Zxilbacteroria
Mn : g__unclassified_p__candidate_division_CSSED10-310
